# A New Topology of the Human Y Chromosome Haplogroup E1b1 (E-P2) Revealed through the Use of Newly Characterized Binary Polymorphisms

**DOI:** 10.1371/journal.pone.0016073

**Published:** 2011-01-06

**Authors:** Beniamino Trombetta, Fulvio Cruciani, Daniele Sellitto, Rosaria Scozzari

**Affiliations:** 1 Dipartimento di Biologia e Biotecnologie “Charles Darwin”, Sapienza Università di Roma, Rome, Italy; 2 Istituto di Biologia e Patologia Molecolari, Consiglio Nazionale delle Ricerche, Rome, Italy; University of Glasgow, United Kingdom

## Abstract

Haplogroup E1b1, defined by the marker P2, is the most represented human Y chromosome haplogroup in Africa. A phylogenetic tree showing the internal structure of this haplogroup was published in 2008. A high degree of internal diversity characterizes this haplogroup, as well as the presence of a set of chromosomes undefined on the basis of a derived character. Here we make an effort to update the phylogeny of this highly diverse haplogroup by including seven mutations which have been newly discovered by direct resequencing. We also try to incorporate five previously-described markers which were not, however, reported in the 2008 tree. Additionally, during the process of mapping, we found that two previously reported SNPs required a new position on the tree. There are three key changes compared to the 2008 phylogeny. Firstly, haplogroup E-M2 (former E1b1a) and haplogroup E-M329 (former E1b1c) are now united by the mutations V38 and V100, reducing the number of E1b1 basal branches to two. The new topology of the tree has important implications concerning the origin of haplogroup E1b1. Secondly, within E1b1b1 (E-M35), two haplogroups (E-V68 and E-V257) show similar phylogenetic and geographic structure, pointing to a genetic bridge between southern European and northern African Y chromosomes. Thirdly, most of the E1b1b1* (E-M35*) paragroup chromosomes are now marked by defining mutations, thus increasing the discriminative power of the haplogroup for use in human evolution and forensics.

## Introduction

Human Y chromosome haplogroups are defined by unique mutations within the male specific region of the Y chromosome (MSY). The lack of recombination in this portion of the genome makes it possible to reconstruct an unequivocal haplogroup phylogenetic tree, which can be related to the geographic distribution of the individual branches of the tree, by an approach known as “phylogeography” [1]. The high genealogical resolution of this system, established by recent advances in mutation-detection technology, and the phylogeographic method have together proven highly informative in tracing patterns of human prehistoric colonization and migrations.

A parsimonious phylogenetic tree for 20 major haplogroups (A-T) representing worldwide Y chromosomal variation was proposed in 2008 [2]. In the present work, we focused on the structure of haplogroup E1b1. Within haplogroup E, which represents the majority of the Y chromosomes found in Africa, E1b1 is the haplogroup which has the greatest geographic distribution. Three lineages, E1b1a (E-M2), E1b1b (E-M215) and E1b1c (E-M329) were included in the genealogy presented by Karafet et al. [2]. To gain a better understanding of the structure of this complicated haplogroup, we performed a high resolution analysis by sequencing, on the average, 45.4 kb in each of 13 E1b1 Y chromosomes (Table S1). Incorporating the information obtained from this analysis into the previously reported tree produced an extensively revised phylogeny for the haplogroup E1b1 resulting in 52 distinct haplogroups.

## Results

A total of 7 new SNPs within the E1b1 clade were discovered and mapped on the Y chromosome tree. In addition, five mutations (M293, V68, V92, V95 and V100) that had been previously described [3]–[5] but not included in the tree reported by Karafet et al. [2], were phylogenetically characterized. Information regarding these 12 SNPs is given in Table 1. Finally, two previously reported mutations, M154 [6] and M281 [7], required changes to their position in the phylogenetic tree as reported by Karafet et al. [2]. Changes to the previous “by lineage” haplogroup names have come about as a result of the incorporation of the new SNPs into the tree. To avoid any confusion, we have referred to the names of previous haplogroups (uninformed by the SNPs here characterized) by adding the term “former” throughout the text.

**Table 1:** List of the phylogenetically characterized polymorphisms in the present study.

SNP	Y-Position^a^	Mutation	Forward Primer	Reverse Primer	RefSNP ID	Reference
M293	22744939	T to G	GATATTAGTATTGAAGAAACCAG	GCTGGCTAATACTTCCACAGAG	rs9341316	[3]
V16	6851471	C to T	GGTTCAGAATCCTCTGGCACTA	ACAAGAGTTACAAGACCGGGAA	-	present study
V23	6860272	C to A	ATAGCTCATGCTGTTTCTGGT	ACACAGCCAGGATTTCTACCTAGTC	-	present study
V38	6818291	C to T	ATTATTTGAATGCAAGTGGGGA	TGAAACTGAATTAAAGGAAGGTGG	rs768983	present study
V39	16282382	C to G	TTAAAATGGGACCACCAAGAGT	TGCAGAGAGCAAAACTAAATGAA	-	present study
V42	16437344	G to A	CTATAACTGCCTTTCTGCAACATG	TCCCTGCATTTTCTACTCTCAGT	-	present study
V43	16691822	G to T	TGAAGGAGATTTAATTGGGGTAGA	TTTCCATACCATGCCTTTCTTT	rs73621792	present study
V68	17664771	A to C	CAACTGAAAATCAGAACTTTGG	GTGGATCACGAGGTCAGG	-	[4]
V92	4873798	C to T	TCTCACTTTCCCCATCCAGA	AGTATTTTATTTTTCCCAAACGTAGC	-	[5]
V95	5274968	C to G	TGAAGTGACTGATCTCGTTTCAT	TTGTTAGCCAGAATGGTCTCA	-	[5]
V100	5335763	G to A	AATGCTCCTGGTAAATGTTTCT	GCATTTCCTGTTGCCTTT	-	[5]
V257	14484546	C to T	CCTCAGGTGGTCATTGCTCTA	CAACAGGAGAAAAGGTGAGAAAC	-	present study

a Postion according to the February 2009 human Y-chromosome reference sequence (GRCh37).

**Figure 1:**
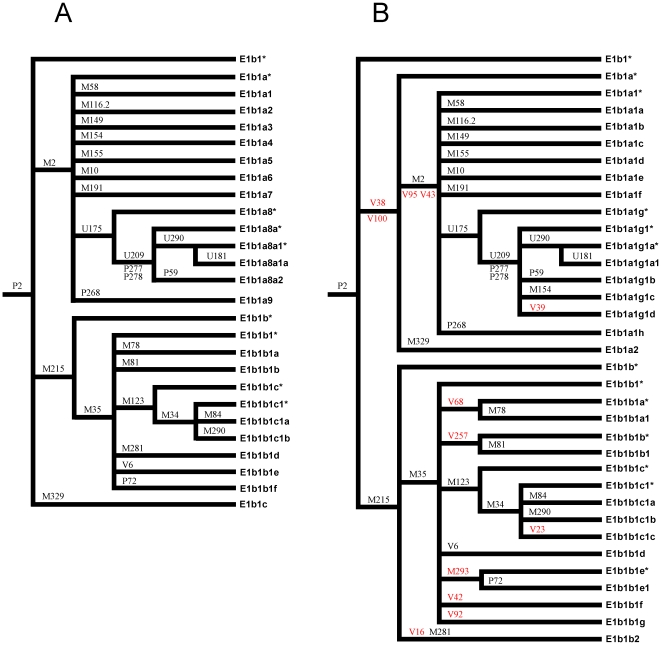
Phylogeny of the haplogroup E1b1 without (A) and with (B) the SNPs phylogenetically characterized in this study (in red). A “by lineage” nomenclature system [21] was used to name the haplogroups. For the sake of clarity, the internal structure of haplogroups E-M191 (six sub-haplogroups), E-M78 (11 sub-haplogroups) and E-M81 (3 sub-haplogroups) is not shown. Paragroups E1b1*, E1b1a* and E1b1b* were never observed in our data set (more than 2,000 African Y chromosomes, data not shown). Note that the mutations M154 and M281 have been repositioned (B) with respect to the previously published phylogeny (A); however, the possibility that M154 and M281 are recurrent mutations cannot be excluded. The positions of the mutations M116.2, P268, P269, M84 and M290 in relation to the newly characterized mutations have not been resolved because of the lack of positive control DNAs.

The phylogeny of the haplogroup E1b1 without (A) and with (B) the newly characterized SNPs is shown in Figure 1. Two SNPs (V43 and V95) turned out to be phylogenetically equivalent to the previously characterized mutation defining haplogroup E1b1a1 (E-M2, former E1b1a). However, incorporating the remaining ten mutations into the tree resulted in important changes compared with the previously published phylogeny.

The tripartite structure of E1b1 has now been resolved by virtue of the new markers V38 and V100, which combined haplogroup E-M2 (former E1b1a) with E-M329 (former E1b1c) into the haplogroup E-V38 (E1b1a). Within this haplogroup, the M154 marker was repositioned to the E-U209 clade. Nine chromosomes out of 95 E-U209*(x U290, P59) turned out to carry the derived state at M154. In addition, a new lineage was found to be defined within the E-U209 clade by the newly discovered V39 mutation. Two among 86 E-U209*(xU290, P59, M154) sub-Saharan chromosomes were found to have this mutation.

The V68 mutation was recently reported to be phylogenetically equivalent to M78 in a sample of 239 African chromosomes [4]. Here, undifferentiated paragroup E-V68*(xM78) chromosomes were observed in 3 among 9 E-M35* previously reported chromosomes from Europe [8]. A newly discovered mutation, V257, combined all the E-M81 and a subset (4/9 from Europe, 1/1 Marrakesh Berber and 1/1 Oromo from Kenya) of the E-M35* chromosomes reported by Cruciani et al. [8]. The V23 mutation was found to mark a new lineage within the E-M34 clade. Two out of 16 E-M34 Y chromosomes which had been previously observed in Africa [8] turned out to carry this mutation. The mutation M293 mutation [3] was shown to be positioned upstream of the P72 marker (Figure 1), which defines the E1b1b1f lineage in the tree by Karafet et al. [2]. All the sixteen Y chromosomes from southern Africa and 4/19 Y chromosomes from eastern Africa described by Cruciani et al. [8] as belonging to paragroup E-M35* turned out to carry the M293 mutation. The E-M35* undifferentiated state of two Jews and one Amhara from Ethiopia previously reported [8] has now been resolved by two mutations (V42 and V92, respectively), that identify two additional clades within the E-M35 haplogroup. Finally, we have found that M281 does not define a separate sub-lineage within E-M35; rather it is phylogenetically indistinct from the newly discovered V16 mutation and marks all the five E-M215* chromosomes reported by Cruciani et al. [4].

In conclusion, incorporating the newly characterized mutations into the E1b1 haplogroup, has led to a total of 52 lineages. This compares with the 44 lineages on the tree by Karafet et al. [2].

## Discussion

Haplogroup E1b1 which is characterized by a high degree of internal diversity is the most represented Y chromosome haplogroup in Africa. Here we report on the characterization of 12 mutations within this haplogroup, eleven of which were discovered in the course of a resequencing and genotyping project performed in our laboratory. There are several changes compared to the most recently published Y chromosome tree [2]. Haplogroup E1b1 now contains two basal branches, E-V38 (E1b1a) and E-M215 (E1b1b), with V38/V100 joining the two previously separated lineages E-M2 (former E1b1a) and E-M329 (former E1b1c). Each of these two lineages has a peculiar geographic distribution. E-M2 is the most common haplogroup in sub-Saharan Africa, with frequency peaks in western (about 80%) and central Africa (about 60%). The same haplogroup is also present in North Africa, although at a lower frequency (usually below 10%) [9]–[11]. Haplogroup E-M329, on the other hand, was observed almost exclusively in eastern Africa [10], [12 and R.S. unpublished data], where E-M2 is virtually absent. The second basal branch of E1b1, E-M215, has a broad geographic distribution from southern Europe to northern and eastern Africa where it has been proposed to have originated [8]. The new topology here reported has important implications as to the origins of the haplogroup E1b1. Using the principle of the phylogeographic parsimony, the resolution of the E1b1b trifurcation in favor of a common ancestor of E-M2 and E-M329 strongly supports the hypothesis that haplogroup E1b1 originated in eastern Africa, as previously suggested [10], and that chromosomes E-M2, so frequently observed in sub-Saharan Africa, trace their descent to a common ancestor present in eastern Africa.

Within E-M35, there are striking parallels between two haplogroups, E-V68 and E-V257. Both contain a lineage which has been frequently observed in Africa (E-M78 and E-M81, respectively) [6], [8], [10], [13]–[16] and a group of undifferentiated chromosomes that are mostly found in southern Europe (Table S2). An expansion of E-M35 carriers, possibly from the Middle East as proposed by other Authors [14], and split into two branches separated by the geographic barrier of the Mediterranean Sea, would explain this geographic pattern. However, the absence of E-V68* and E-V257* in the Middle East (Table S2) makes a maritime spread between northern Africa and southern Europe a more plausible hypothesis. A detailed analysis of the Y chromosomal microsatellite variation associated with E-V68 and E-V257 could help in gaining a better understanding of the likely timing and place of origin of these two haplogroups.

Thanks to the newly characterized mutations, the large majority (34/45) of the chromosomes previously assigned to paragroup E-M35* [8] are now defined by unique mutations (Table S2). These findings will be of importance to those with research interests in human evolution [17]–[18] and forensic issues [19], [20].

## Materials and Methods

This study was approved by the “Policlinico Umberto I, Sapienza Università di Roma” Ethical Committee (protocol number 1016/2010, according to the DM 15/7/1997 and following). The data were analyzed anonymously.

DNA samples came from collections of the Authors, and haplogroup information is as reported [4], [8]. DNAs were obtained from a total of 174 individuals from each of the following haplogroups E-M2* (3), E-U209* (95), E-V39, E-M215* (5), E-M35* (45), E-M78, E-V257*, E-M34*(18), E-V23, E-M293*, E-V42, E-V92 and E-V16.

### Amplification and Sequencing

Overall, 45,4 Kb were sequenced on the average for each of 13 unrelated Y chromosomes (Table S1).

We designed polymerase chain reaction (PCR) and sequence primers on the basis of the Y-chromosome sequence reported in the February 2009 assembly of the UCSC Genome Browser (http://genome.ucsc.edu/) using Primer3 software (http://frodo.wi.mit.edu/primer3/). Sequencing templates were obtained through PCR in a 50-µl reaction containing 50 ng of genomic DNA, 200 µM each deoxyribonucleotide (dNTP), 2.5 mM MgCl_2_, 1 unit of Taq polymerase, and 10 pmoles of each primer. A touch-down PCR program was used with an annealing temperature decreasing from 62°C to 55°C over 14 cycles, followed by 30 cycles with an annealing temperature of 55°C.

Following DNA amplification, PCR products were purified using theQIAquick PCR purification kit (Qiagen, Hilden, Germany). Cycle sequencing was performed using the BigDye Terminator Cycle Sequencing Kit with Amplitaq DNA polymerase (Applied Biosystems, Foster City, CA) and an internal or PCR primer. Cycle sequencing products were purified by ethanol precipitation and run on an ABI Prism 3730XL DNA sequencer (Applied Biosystems). Chromatograms were aligned and analyzed for mutations using Sequencher 4.8 (Gene Codes Corporation, Ann Arbor, MI).

Mapping and genotyping of the new mutations were performed by using a total of 174 DNA samples classified as indicated above.

## Supporting Information

Table S1 **Summary information for 13 Y chromosomes analyzed by sequencing.**
(XLS)

Table S2 New haplogroup/paragroup assignment for 45 E-M35*(xM78,M81,M123,V6) previously reported chromosomes.
(XLS)
